# Supplementary material for: Does Exercise-Induced Hypoalgesia Depend on Exercise Duration?
Source: Biology (Basel). 2023 Jan 30;12(2):222. doi: 10.3390/biology12020222 (PMC9953562; doi:10.3390/biology12020222)
Supplement: Supplementary file 1 [file biology-12-00222-s001.zip › biology-2080438-supplementary.pdf]

Table S1. Number of responders and non-responders with respect to the three exercise sessions conducted. Data are presented as the absolute number of responsive and non-responsive subjects for each landmark evaluated.

	30 Min Exercise Session		45 Min Exercise Session		60 Min Exercise Session	
	Responder	Non-Responder	Responder	Non-Responder	Responder	Non-Responder
**Ankle left**	14	22	8	28	10	26
**Ankle right**	14	22	11	25	8	28
**Knee left**	12	24	8	28	9	27
**Knee right**	10	26	6	30	7	29
**Elbow left**	11	25	15	21	13	23
**Elbow right**	13	23	11	25	6	30
**Sternum**	14	22	10	26	6	30
**Forehead**	8	28	9	27	4	32
